# Nanostructured Lipid Carriers Containing Acridine Derivatives in the Application of Sonodynamic Therapy for the Treatment of Breast Cancer

**DOI:** 10.1021/acsomega.5c11337

**Published:** 2026-02-03

**Authors:** Kammila Martins Nicolau Costa, Mariana Rillo Sato, Ricardo Olimpio de Moura, Anthony P. McHale, John F. Callan, João Augusto Oshiro-Junior

**Affiliations:** † Postgraduate Program in Development and Technological Innovation in Medicines, 28097 Federal University of Paraíba, João Pessoa 58051-900, Paraíba, Brazil; ‡ Pharmaceutical Sciences Postgraduate Program, Center for Biological and Health Sciences, State University of Paraíba, Av. Juvêncio Arruda, S/N, Campina Grande 58429-500, PB, Brazil; § 2596 Ulster University, Coleraine BT52 1SA, Northern Ireland, U.K.

## Abstract

Through sonosensitizing agent activation, sonodynamic therapy (SDT) generates an imbalance of reactive oxygen species (ROS) that is potentially damaging to target cells. Acridine derivatives (AD), such as AMTAC compounds (01, 02, 18, 22), present properties of sonosensitizing agents for use in SDT. This work aims to formulate and characterize nanostructured lipid carriers (NLC) containing AD for the application of SDT as a low-invasive breast cancer (BC) treatment. DLS, ELS, SOSG assay, MTT assay, *in vitro* release study, and *in vivo* efficacy techniques were applied. DLS and ELS showed a size range of 107.03 to 119.96 nm, zeta potential of −14.31 to −4.12 mV, and a polydispersity index of 0.17 to 0.26. The SOSG assay demonstrated that AMTAC 02 generated the highest amount of ROS (282%). The combination of SOSG and the MTT results, which showed cell viability <40% for all the compounds, resulted in the selection of AMTAC 02 to proceed with further studies. The NLC+AMTAC 02 formulation showed a modified release profile compared to pure AMTAC 02. The *in vivo* assay demonstrated that the system has a tumor inhibition potential 6 times greater than the untreated group. The data suggested that NLC-AMTAC 02-mediated SDT could represent a promising treatment modality for BC.

## Graphical abstract

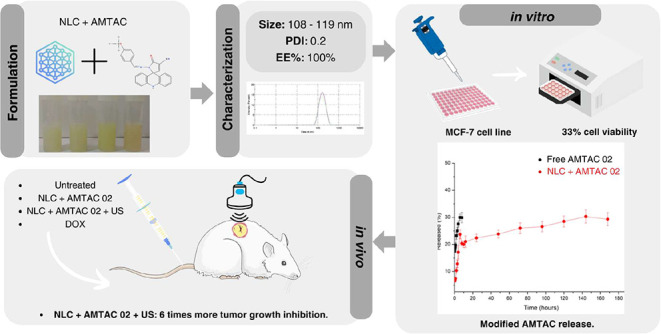

## Introduction

Breast cancer (BC) is the second most common type of cancer affecting women, being surpassed only by skin cancer.^1–3^ Worldwide, the pandemic caused by COVID-19 has made treatment and diagnosis even more difficult, directly affecting the number of diagnosed cases linked to BC, as well as the number of deaths related to the disease, which, due to the situation, reached around 685,000 cases in 2020. In addition, it is estimated that by 2040, the number of cases could increase by 40%, while the number of lethal cases could rise from 685,000 to 1 million.^4,5^

Although there are various approaches to treating BC (surgery, radiotherapy, chemotherapy, hormonal therapy, and immunotherapy, combined with conventional drugs), the adverse effects and low specificity of the approaches used limit therapeutic efficacy, leading to a high level of patient adverse effects and, in extreme cases, treatment abandonment.^6–8^ In this sense, new alternatives and minimally invasive treatment modalities such as sonodynamic therapy (SDT) have been investigated for application in the field of oncology.^9^

SDT consists of activating a sonoreactive molecule or sonosensitizing agent (SA) using ultrasound (US), which, through the cavitation mechanism, will interact with molecular oxygen and cause cell death by exacerbated generation of reactive oxygen species (ROS).^9–13^ While the mechanism for ROS generation is not completely understood, several reports have suggested a potential role for sonoluminescence in activating the sensitizer in a manner similar to photodynamic therapy (PDT), whereby one of the main disadvantages is the concentration of molecular oxygen present in the tumor.^9–13^

However, unlike PDT, SDT is capable of achieving a deeper level of tissue penetration, currently being applied to clinical and preclinical studies in a variety of cancers.^9–13^ Thus, the proposal to apply a system that responds to US stimulation by seeking to release AS to increase ROS generation may provide a more selective, effective, and safer approach, reducing the risks of systemic toxicity.^13,14^

Acridines, which are a class of molecules that have a pyridine ring fused to two benzene rings, present antifungal and antibacterial pharmacological activities; however, their suggested application is particularly focused on anticancer therapy.^15–19^ The biological activities of its derivatives, especially 3-(acridin-9-yl)-2-cyano-N-(4-methoxybenzylidene)-acrylohydrazide (AMTAC), have been explored, as well as the possibility of being applied as sonosensitizing agents.^17,18,20–22^

The AMTAC molecule can undergo structural modifications, resulting in various derivatives with differing properties. Modifications in these derivatives occur through synthesis, where initially different aromatic aldehydes can be condensed with a 2-cyanoacetohydrazide portion, forming different intermediates that are subsequently condensed with acridine aldehyde, resulting in different spiro acridine derivatives, which comprise the AMTAC family.^15,23,24^ However, acridine derivatives such as AMTAC have a major limitation linked to solubility, motivating us to develop new nanosystem options to improve or solve this problem.^15,23,24^

Nanostructured lipid carriers (NLC) are one of the options among nanosystems that can help, as well as ensure the compound’s protection.^7,8,25,26^ These are systems with an irregular lipid matrix, capable of storing a large concentration of drugs with less risk of expulsion, as is likely to happen with solid lipid particles; however, they have a disadvantage in terms of long-term storage, requiring certain strategies, such as presentation in extemporaneous pharmaceutical form. Drugs approved by the Food and Drug Administration (FDA) for the treatment of cancer and featuring lipid nanoparticles (LNP) exploit liposome technology, with several formulations commercially available (Doxil, Myocet, DaunoXome, Onyvide).^27–30^

The global acceptance of LNP through the application of liposome-loaded vaccines against COVID-19, produced by major industrial centers, has contributed to the growing research landscape in this area.^31^ LNP has been applied for a similar purpose, this time for the delivery of siRNA in the drug Onpattro, approved by the FDA in 2018. Used for the treatment of hereditary transthyretin amyloidosis, the presence of nanoparticles in the formulation has improved the compound’s pharmacokinetics and pharmacodynamics, as well as making this type of nanoparticle less expensive, more stable, and easier to produce on a large scale when compared to liposomes, for example.^27–30^

Thus, considering the minimally invasive nature of SDT and the promising responses found in the literature for the treatment of breast cancer, seeking to reduce or eliminate severe adverse effects, the work described here seeks to explore the possibility of using NLC as a vehicle for acridine derivatives and demonstrate their efficacy in SDT-mediated treatment of BC.

## Materials and Methods

### Preparation of Nanostructured Lipid Carriers

The NLC formulation was prepared using the emulsification-evaporation methodology proposed by Sato et al.,^32^ with modifications. The lipid phase of the system was composed of 2.05% (w/w) triglycerides of capric/caprylic acids (TGACC) (MedChemTronica AB, Sweden), 2.05% (w/w) polyoxyethylene stearate 40 (PS40, Sigma-Aldrich, São Paulo, Brazil), as well as 0.88% (w/w) ethoxylated hydrogenated castor oil 40 (Sigma-Aldrich, United Kingdom) and the different AMTACs (synthesized by the Laboratory for Drug Development and Synthesis (LDSF), Universidade Estadual da Paraíba, Brazil). The aqueous phase was formed by 3.5% (w/w) Pluronic F127 (Sigma-Aldrich, United Kingdom) and ultrapurified water. Both phases were heated and kept under constant stirring in magnetic stirrers (Stuart SB 162–3) until the temperature reached 70 ± 5 °C. The aqueous phase was poured into the lipid phase under agitation, thus forming a pre-emulsion. The system was sonicated using an ultrasonic cell disrupter (Sonics Vibra Cell) with an amplitude of 35% for a total of 4 cycles of 1 min, with an interval of 30 s each.

The acridine derivative compounds AMTAC 01, AMTAC 02, AMTAC 18, and AMTAC 22 were added to the lipid phase portion at a concentration of 0.5%, giving rise to NLC + AMTAC (Table S1).

### Determination of Mean Hydrodynamic Diameter (d nm)

The four formulations containing AMTAC incorporated into the NLC (NLC + AMTAC 01, 02, 18, and 22) and the blank carriers were subjected to dynamic light scattering (DLS) to analyze the average hydrodynamic diameter and polydispersity index, as well as the zeta potential of the relevant formulations, using the Malvern Zetasizer Nano-ZS Zen3600 (Malvern Panalytical Ltd., United Kingdom). The measurements were carried out in triplicate at 25 °C, 173° beam angle, using disposable cuvettes. The results were expressed as the mean and standard deviation of the values obtained.

### Encapsulation Efficiency (EE%)

The encapsulation efficiency of the different AMTAC compounds incorporated at different concentrations (0.1, 0.5, 1, and 3%) in relation to the lipid mass of the NLC was obtained after centrifugation at 5000 rpm for 30 min. The supernatant was then filtered using a 0.45 μm cellulose membrane (Minisart NML, UK) to separate the encapsulated drug from the nonencapsulated precipitate. The content of AMTAC incorporated into the NLC was quantified using a Varian Cary Eclipse Fluorescence Spectrophotometer at 435 nm with excitation at 370 nm (slit width of 10 nm) and determined using eq 1:

1 $$\mathrm{EE}\%=\frac{M1}{\mathrm{MT}}\times 100$$

where “EE%” is the encapsulation efficiency percentage; “M1” is the intensity of the AMTAC incorporated into the NLC, and “MT” is the theoretical intensity of the AMTAC used during the preparation of the relevant NLC formulation.

### SOSG Assay

To carry out the assay, 0.25 mL of the four different AMTAC compounds (0.125 mg/mL) were added to a solution of singlet oxygen sensor green (SOSG 2.5 μM, Invitrogen, UK) in 1.75 mL of methanol, followed by exposure to US (Sonidel Ltd., Ireland) for 30 min (power density 3 W cm^–2^, frequency 1 MHz and pulse repetition frequency 100 Hz) using a Sonidel SP300 ultrasound generator (Dublin, Ireland). SOSG fluorescence intensity (after excitation at 505 nm) was taken at 5 min intervals (Cary Eclipse Fluorescence Spectrophotometer Varian) at 525 nm. This protocol was applied to the AMTAC (01, 02, 18, and 22) and AMTAC (01, 02, 18 2 22) + US groups. SOSG was expressed as a percentage and calculated using eq 2:

2 $$\%\mathrm{SOSG}=\frac{\mathrm{Isample}-\mathrm{Itime}\;0}{\mathrm{Itime}\;0}\times 100$$

where “Isample” is the average of the sample’s intensity, and “Itime 0” is the average of the sample’s intensity measured at time 0.

### Cell Culture

MCF-7 and MDA-MB-468 breast cancer cells were purchased from the cell line bank (ATCC Cell Bank, Middlesex, United Kingdom) and cultured in Dulbecco’s Modification of Eagle’s Medium (DMEM) low glucose (Sigma-Aldrich, United Kingdom) supplemented with 1% penicillin-streptomycin and 10% fetal bovine serum (FBS), and DMEM high glucose with GlutaMAX (Sigma-Aldrich, United Kingdom) supplemented with 10% fetal bovine serum (FBS), and 1% penicillin-streptomycin. The cells were cultured in T-25 flasks and incubated at 37 °C in a humidified atmosphere containing 5% CO_2_.

### MTT Assay

The cell concentration used in the assay was 5 × 10^4^ cells/mL, where 100 μL of suspension was deposited in each well of a 96-well plate and incubated for 24 h at 37 °C and a humidified atmosphere containing 5% CO_2_. Cells were treated with 100 μL of each of the following 20 groups: NLC + AMTAC 01, 02, 18, and 22 in three different concentrations (6.6, 13.2, and 66 μM) without US application, and only the 66 μM exposed to US (a frequency of 1 MHz, a power density of 3 W cm^–2^, a duty cycle of 30 s, and a pulse frequency of 100 Hz), as well as negative, positive and medium controls (Table 1). After 24 h of incubation, all treatments were removed, 100 μL of media was added, and 10 μL of MTT solution (5 mg/mL of PBS) was added to each well and incubated for 3 h at 37 °C. All the material was removed from the wells for the subsequent addition of 200 μL of DMSO to dissolve the formazan crystals.^33^ Spectrophotometric reading was carried out on a microplate reader (FLUOstar, BMG Labtech, Ortenberg, Germany) at an absorbance of 570 nm. The experiment was conducted in triplicate. The detailed scheme of the treatments can be seen in Table 1.

**Table 1:** MCF-7 Cell Treatment System During the MTT Assay^a^

**treatment**	**concentration (μM)**	**ultrasound (seconds)**
NLC + AMTAC 01	6.6	
NLC + AMTAC 01	13.2	
NLC + AMTAC 01	66	
NLC + AMTAC 01	66	30
NLC + AMTAC 02	6.6	
NLC + AMTAC 02	13.2	
NLC + AMTAC 02	66	
NLC + AMTAC 02	66	30
NLC + AMTAC 18	6.6	
NLC + AMTAC 18	13.2	
NLC + AMTAC 18	66	
NLC + AMTAC 18	66	30
NLC + AMTAC 22	6.6	
NLC + AMTAC 22	13.2	
NLC + AMTAC 22	66	
NLC + AMTAC 22	66	30
NLC control		
NLC control		30
DOX control	46	
DMEM*		

a Nanostructured lipid carriers (NLC); Doxorubicin (DOX); Dulbecco’s Modification of Eagle’s Medium (DMEM), Control applied to the equation (*).

Cell viability was expressed as a percentage inhibition in relation to the controls, calculated using eq 3:

3 $$\%\;\mathrm{cell}\;\mathrm{viability}=\frac{\mathrm{abs}\;\mathrm{of}\;\mathrm{sample}}{\mathrm{abs}\;\mathrm{of}\;\mathrm{control}}\times 100$$

where “control abs” is the absorbance of the untreated cells and “sample abs” is the absorbance of the treated cells.

### Calibration Curve to Determine AMTAC Concentration in NLC

The analytical curve was determined by Varian Cary Eclipse Fluorescence Spectrophotometer at 435 nm with excitation at 370 nm (slit width of 10 nm). The diluent employed consisted of phosphate-buffered saline (PBS) (Sigma-Aldrich, United Kingdom) and dimethyl sulfoxide (DMSO) (Sigma-Aldrich, United Kingdom) (50:50%). Linearity was determined using a 1 mM stock solution of AMTAC 02, from which four concentrations of 2, 4, 6, and 8 μM were prepared. The limits of detection and quantification were determined.

### Release Assay

With the sink condition guaranteed at 4x, the release test was carried out using the dialysis membrane method in beakers. Aliquots of 01 mL of AMTAC 02 and NLC + AMTAC 02 were inserted into 25 mm flat-width dialysis tubes (D9777–100FT, Sigma-Aldrich, United Kingdom) with a cutoff of 14 000 Da, immersed in a beaker containing 50 mL of PBS and DMSO. The systems were kept under constant agitation (Stuart SB 162–3 x3) at 500 rpm, 37 °C, and protected from light throughout the procedure. An aliquot of 3 mL was taken at predetermined times of 0.25, 0.5, 0.75, 1, 2, 3, 4, 6, 8, 10, 12, 24, 48, 72, 96, 120, 144, and 168 h. The fluorescence was analyzed in a Varian Cary Eclipse Fluorescence Spectrophotometer at an emission wavelength of 435 nm, using an excitation of 370 nm (slit width of 10 nm), using the parameters of the calibration curve. The experiment was carried out in triplicate, and the data plotted correlated time versus the percentage of AMTAC released.

### *In Vivo* Efficacy

All animals employed in this study were treated humanely and in accordance with licensed procedures under the UK Animals (Scientific Procedures) Act 1986. Local ethical approval was obtained from the institutional Animal Welfare and Ethical Review Board (AWERB).

The human breast cancer cell line, MDA-MB-468, was used to generate tumors in 20 BALB/c mice aged 6–8 weeks by injecting 100 μL of cells (5 × 10^6^ cells/animal in Matrigel in the rear dorsum of recipient animals. The tumors grew until they became palpable, for approximately 1–2 weeks.

The animals were randomly coordinated according to the size of the tumor presented once they had an average volume of 65 mm^3^. To quantify the percentage of tumor growth, 4 groups of 5 female BALB/c mice were determined: (a) untreated animals; (b) NLC + AMTAC 02; (c) NLC + AMTAC 02 + US; (d) doxorubicin (DOX) (2 mg/kg).

The animals were anesthetized by inhalation of 2% (v/v) isofluorane (ISOFLO) stream in a 100% O_2_ carrier supplied at a flow rate of 2 L min^–1^. The treatment protocol consisted of intravenous administration of the formulation via the caudal vein (100 μL) on days 0, 3, 6, and 10, while DOX was administered once a week. In the relevant group, ultrasound was directly applied to the tumor during injection (3.5 min) and 30 min after treatment, using the Sonidel SP300 sonoporator (3.5 W cm^–2^, 1 MHz, 30% duty cycle and PRF = 100 Hz; PNP = 0.48 MPa; MI = 0.48).

After the treatment, the animals were allowed to recover from the anesthesia, and the tumor volume and body weight were recorded. The readings were taken on days 0, 3, 5, 7, 10, 12, 13, and 17 after treatment using Vernier calipers, by measuring the geometric volume. At the end of the experiment, the animals were sacrificed by exposure to carbon dioxide gas in a rising concentration.

### Statistical Analysis

The results presented in this study were statistically analyzed through analysis of variance (ANOVA), applying multiple comparisons using the Tukey method, with GraphPad Prism 8.0.1 software. The level of significance adopted was *p* < 0.05.

## Results and Discussion

### Preparation of NLCs and Determination of Mean Hydrodynamic Diameter (d.nm)

Using the emulsification-evaporation technique, the four different AMTAC molecules incorporated into the NLC were analyzed for their organoleptic characteristics, as shown in Figure 1.

**Figure 1:**
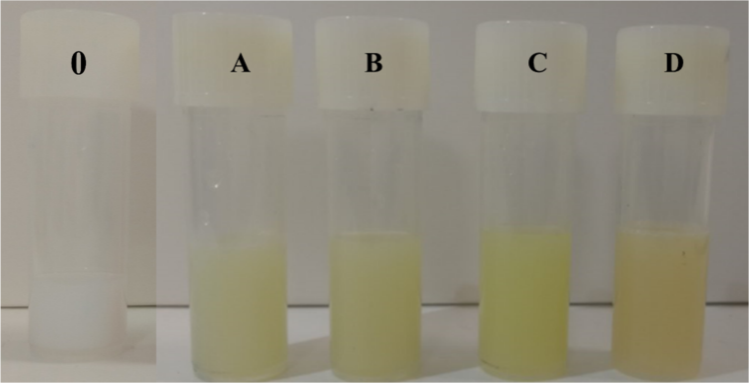
Visual appearance of nanostructured lipid carriers blank (**0**), containing AMTAC 01 (A), 02 (B), 18 (C), and 22 (D).

The addition of the sonosensitizing agent to the system favored the appearance of an opaque, yellowish color pattern from the orange-colored powders of the compound, so the four NLCs containing AMTAC showed a liquid consistency and maintained their integrity with no indication of visual instability such as flocculation, cremation, coalescence, or phase separation.

Subsequently, the NLC with and without AMTAC were assessed for physical stability using the DLS technique. The results shown in Table 2 refer to the average diameter (nm) and polydispersity index (PDI) of the NLCs, as well as the zeta potential (ZP) results of the particles.

**Table 2:** Results of Average Diameter, Polydispersity Index, and Zeta Potential of NLCs Containing the Four Different AMTAC Molecules and Blank NLC^a^ ^,^ ^b^

**nanoparticles**		**average size (d.nm)**	**PDI**	**ZP (mV)**
before lyophilization	NLC	118.05 ± 0.49	0.23 ± 0.030	–9.20 ± 1.20
	NLC + AMTAC 01	117.80 ± 0.28	0.24 ± 0.060	–10.00 ± 0.25
	NLC + AMTAC 02	112.60 ± 0.91	0.25 ± 0.010	–12.80 ± 2.93
	NLC + AMTAC 18	107.03 ± 2.50	0.23 ± 0.009	–14.31 ± 3.40
	NLC + AMTAC 22	115.96 ± 1.90	0.24 ± 0.090	–11.38 ± 2.08
after lyophilization	NLC	119.96 ± 2.00	0.17 ± 0.009	–7.70 ± 1.20
	NLC + AMTAC 01	117.60 ± 0.05	0.20 ± 0.005	–8.25 ± 0.03
	NLC + AMTAC 02	111.80 ± 1.00	0.22 ± 0.002	–9.55 ± 0.43
	NLC + AMTAC 18	109.90 ± 0.90	0.26 ± 0.006	–4.12 ± 0.26
	NLC + AMTAC 22	108.05 ± 0.65	0.218 ± 0.011	–7.75 ± 0.71

a Data is expressed as mean ± SD (*n* = 3).

b Nanostructured lipid carriers (NLC), Polydispersity index (PDI), zeta potential (ZP).

The particle size analysis provided is an important criterion for verifying the stability of the system, since it can influence the biodistribution, cellular uptake, and release rate of the sonosensitizing compound, and can determine important information, such as the *in vivo* performance of nanosystems, especially when related to tumors.^34^

The size range of the particles must be low enough to avoid capture by immune system cells and large enough to avoid premature elimination through the kidney. Solid tumors exhibit atypical, leaky vasculature that does not occur in normal tissues. Intravascular pores have a size range from 100 to 780 nm, thus allowing the penetration and retention of nanoparticles, otherwise known as the enhanced permeability and retention effect (EPR).^35–37^ Therefore, particles in the range of 100–200 nm are ideal if the EPR is to be exploited for passive accumulation of the relevant nanoparticulate formulation.^38^

Table 2 shows that the average diameter values of the blank NLC before and after lyophilization (118.05 and 119.96 nm, respectively) do not differ significantly from those of the NLC loaded with AMTAC 01, 02, 18, and 22, in which the average diameter range varied between 108.05 and 117.80 nm even after lyophilization. These data demonstrate good physical stability since the particles before and after lyophilization exhibit similar characteristics.

According to studies in the literature, nanoparticles with a size between 100–150 nm can have an improved pharmacological profile, prolonged blood circulation (longer half-life), and advantages over passive targeting of the target region.^35–37^ Thus, the results obtained for the formulations in the current study are in the optimum range for exploiting the EPR effect, precluding premature elimination by the kidney and avoiding uptake by immune system cells.

The PDI results showed values close to 0.2 for the blank NLC and the samples containing the AMTAC molecules, with no significant differences before and after lyophilization, indicating low polydispersity/homogeneity of the particle size distribution. This is due to the fact that the PDI has an acceptable range of values from 0 to 1, where preferably a PDI less than or equal to 0.2 suggests a low polydispersity of the particles and greater than or equal to 0.4 indicates a high standard deviation or a polydisperse system.^26,39,40^

As a result, the application of the lyophilization technique has proved to be an advantageous technological option for preserving stability in the system, preventing degradation, and increasing the product’s shelf life in the long term, when compared to solutions, for example, from the application of an extemporaneous formulation.^41^

Another important parameter to observe for nanosystems is the ZP, which represents the electrical charge on the surface of the particles and is directly related to the physicochemical stability of lipophilic systems. The higher the ZP value, the more likely it is that there will be high electrostatic stability between the particles, since the possibility of aggregation caused by the application of repulsive forces will be minimal.^26,40^

The ZP values found in the formulations (blank NLC and NLC + AMTAC) vary between −14.31 and −4.12 mV with no significant changes before lyophilization and after lyophilization. This could mean that the low ZP value is due to the coating of the NLC, leading to a reduction in the electrophoretic mobility of the particles, provided by the nonionic polymer, Pluronic F127, which is considered to be less toxic and irritating.^42^ In addition, the negative charge can be explained by the ionization of groups present in the system components, polyoxyethylene 40 stearate, and capric/caprylic acid triglycerides.^25,43^

### Encapsulation Efficiency (EE%)

To establish the amount of AMTAC encapsulated in the NLC, the EE% data shown in Table 3 were calculated according to eq 1.

**Table 3:** EE% Data in NLC for the Different Types of AMTAC with Concentrations of 0.1, 0.5, 1, and 3%^a^ ^,^ ^b^

	**concentrations (%)**			
**compounds**	**0.1%**	**0.5%**	**1%**	**3%**
AMTAC 01	100 ± 2.30	100 ± 1.00	32,5 ± 0.09	16,7 ± 2.21
AMTAC 02	100 ± 0.20	100 ± 0.80	41,3 ± 1.02	17,2 ± 3.74
AMTAC 18	100 ± 2.00	100 ± 2.20	46,2 ± 3.20	12,5 ± 1.71
AMTAC 22	100 ± 3.10	100 ± 1.80	38,2 ± 1.10	16,2 ± 0.92

a Data is expressed as mean ± SD (*n* = 3).

b Nanostructured lipid carriers (NLC); Percentage of encapsulation efficiency (EE%).

It appears that the difference in structure of the AMTACs did not interfere with the percentage of EE, since there was no significant difference in EE% of AMTAC in the four formulations analyzed. The results showed that the EE% decreased when higher concentrations of AMTAC were employed (1 and 3%), exhibiting values of less than 46%. The 0.1 and 0.5% ranges, on the other hand, showed an EE% index of 100% encapsulation, favoring 0.5%, which corresponds to a concentration of 66 μM, as the concentration indicated for incorporating the AMTAC molecules into the NLC.

### SOSG Assay

To indirectly identify the production of singlet oxygen through the SOSG test, the different acridine derivatives were analyzed without US and with 30 s of US exposure (3 W cm^–2^) (Figure 2), providing a result of the percentage of fluorescence generation as a function of time.

**Figure 2:**
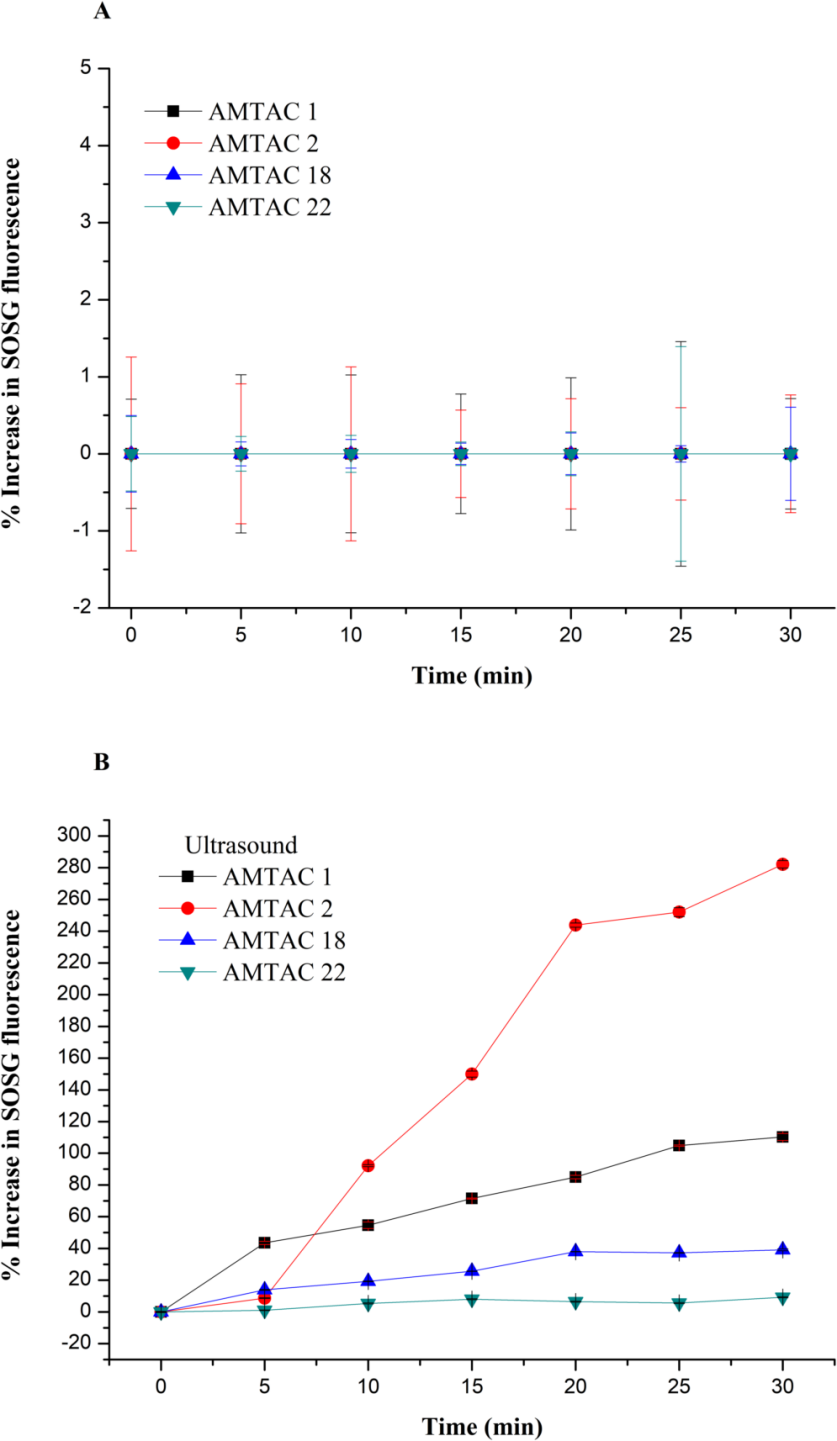
Fluorescence generation percentage curves in the SOSG assay as a function of time for AMTAC 01, 02, 18, and 22, without ultrasound exposure (A) and with ultrasound exposure (B). Data is expressed as mean ± SD (*n* = 3), *p* < 0.05.

The production of fluorescence, in this case, is directly linked to the responsiveness of the SDT due to the lack of a suitable calibration standard for the assay; the exact concentrations of ROS present cannot be quantified.^44^ As a result, it can be seen that the absence of US in the AMTAC compounds (A) did not show SOSG emission and, therefore, ROS generation by the free molecules during the 30 min. However, it can be observed that the fluorescence intensity increases with the duration of ultrasound exposure, which is consistent with tests conducted with porphyrin and phthalocyanine derivatives, once the former is one of the pioneering agents used in the treatment of SDT and PDT.^45–47^

On the other hand, the AMTAC molecules exposed to US (B) showed different degrees of SOSG emission generation, probably due to the presence of the acridine group, which is mainly responsible for the therapeutic effect of these compounds, which interact with the US through acoustic cavitation, favoring the formation of ROS.^48^ At the 5 min mark, there were no significant values for the percentage of SOSG fluorescence emission for AMTAC 02, 18, and 22 (8.69%, 13.89%, and 1.05%, respectively). However, AMTAC 01 showed 54.64% emission in 10 min.

In the case of AMTAC 01, the -H ligand linked to the *para* position of the benzene ring (Figure S1) ends up giving the compound a lower electronic density compared to the other AMTACs and their ligands. However, subsequently, there is a short window of duration in the generation of ROS, which is attributed to their inactivation, through the electronic-vibrational coupling that the C–H bonds, high-frequency oscillators, provide.^49,50^

Between 10 and 30 min of exposure to US, AMTAC 01, 18, and 22 showed a low percentage of SOSG emission initially and increased over time, especially for AMTAC 01, varying from 54.64%, 19.27%, 5.36% to 110.23%, 39.11% and 9.23%, respectively.

The AMTAC 02 and 18 molecules have an –OCH_3_ (methoxy) group in the *para* position, generating a methoxybenzene; however, the latter has another methoxy group in the *ortho* position. Studies show that the presence of this ligand gives these molecules the possibility of greater photogeneration of ROS,^51^ thus corroborating with AMTAC 02, in which from the 10 min range there was an increase in SOSG emission of 92.16%, reaching approximately 282% in 30 min; however, it is possible that the electronic effects of the two methoxyl groups in the same aromatic ring of AMTAC 18 may have influenced a lower fluorescence emission for this compound.

AMTAC 22 has a pyrrole ligand (C_4_H_5_N) attached to the benzene ring, which can cause an increase in the sensitization of the singlet oxygen photosensitizer since its presence leaves it susceptible to conditions directly linked to the temperature and solvents present in the reaction. Researchers have stated that a modification of the pyrrole group, generating its core reactivity attenuation, can allow for a controlled and applicable percentage of ROS generation.^50,52,53^

In addition, the steric hindrance present in AMTAC 18 and 22 molecules, caused by the addition of bulky pairs, can result in a decrease in chemical reactions,^54,55^ as was observed in our results.

Norio et al. analyzed the ability of acridine to generate singlet oxygen and free radicals to study the mechanism proposed by this class of compounds. The results demonstrated significant generation of singlet oxygen and hydroxyl radicals, which are highly reactive and strong oxidizing agents.^56^ Caliskan et al. used ROS generation detection techniques similar to SOSG, and the results showed a high quantum yield, with a significant increase in ROS generation that induced apoptosis in HL60 cells.^57^ Wang et al., suggest that acridine orange has high oxidative activity observed during the damage process triggered by exposure to US,^58^ corroborating the findings presented in the present study.

### The Effect of NLC-AMTAC Formulations on MCF-7 Cell Viability

In these studies, MCF-7 cells were treated with the NLC formulation containing different amounts (low, medium, and high) of acridine derivatives. In addition, cells were treated with each formulation containing the highest concentration of AMTAC together with ultrasound, and the data are shown in Figure 3. As controls, the NLC blank without US and the NLC blank + US with an exposure time of 30 s, DOX (46 μM), DMEM, and US were tested.

**Figure 3:**
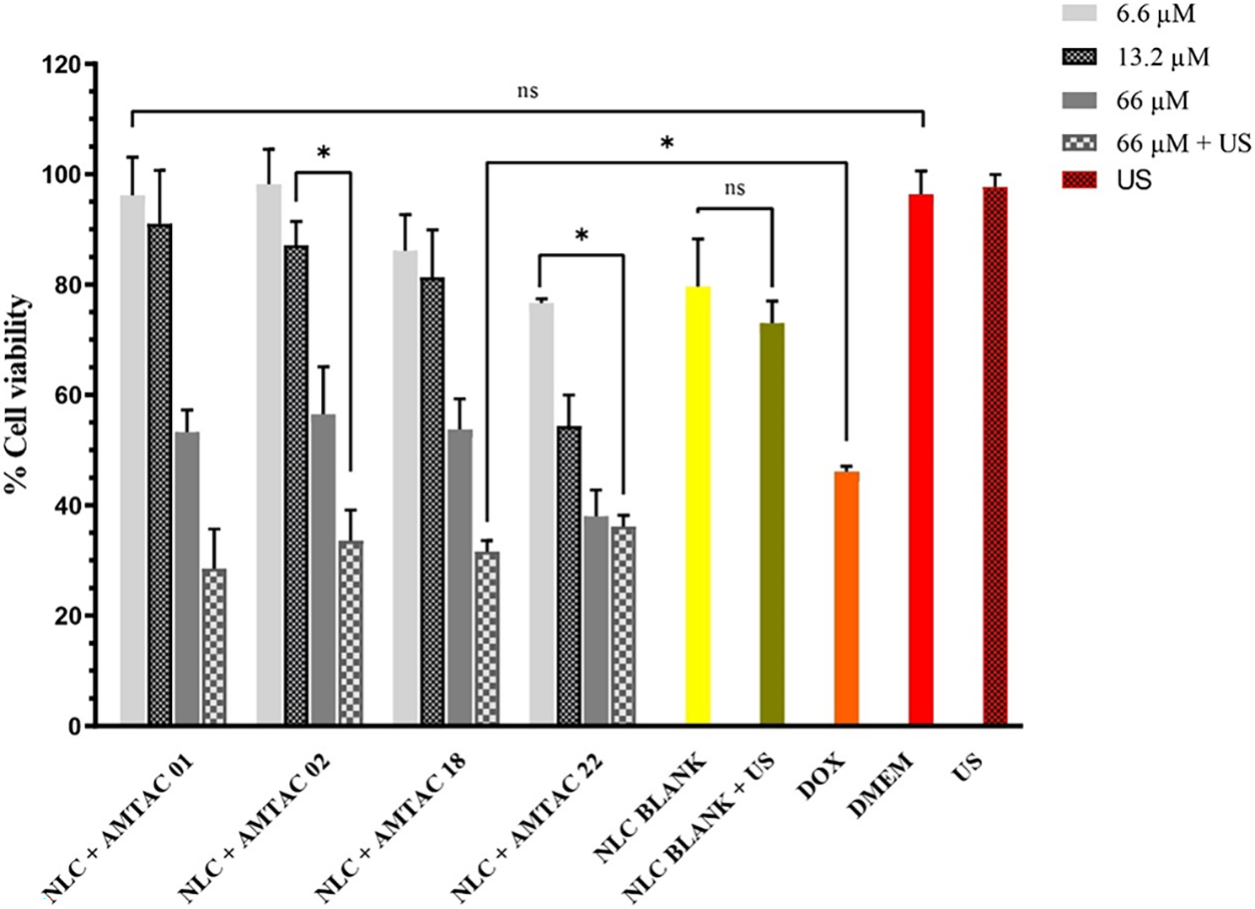
Graph showing the percentage of cell viability for MCF-7 cells treated with AMTAC molecules at high concentration (66 μM), medium concentration (13.2 μM), low concentration (6.6 μM), and high concentration + US, as well as NLC blank and NLC blank + US, and the controls applied. The results are expressed as the mean and standard deviation (SD) of *n* = 3, *p* < 0.05. Note: Nanostructured lipid carriers (NLC); Doxorubicin (DOX); Dulbecco’s Modified Eagle Medium (DMEM); Ultrasound (US); Not significant (ns); Significant (*).

These data demonstrate that the toxicity of each formulation is dose-dependent. The IC_50_ for each formulation was calculated, generating values for AMTAC 01, 02, 18, and 22 of 70.54, 75.43, 72.87, and 40.82 μM, respectively (Table S1). These values suggest greater cytotoxicity for the AMTAC compounds tested when compared to DOX, which, after being analyzed by Ansary et al., demonstrated an IC_50_ of 453 ± 3.1 μM (262.96 ± 1.807 μg·mL^–1^).^59^

The DMEM control group and the US group showed the expected cell viability of over 95%, demonstrating that the US parameters had no effect on cell viability. Treatment with blank NLC in the presence and absence of US showed a variation of 6%, suggesting that the NLC had a low degree of toxicity and confirming that the US parameters employed in these experiments had very little effect on cell viability.

In terms of high concentration (66 μM), the results were significant for NLC + AMTAC 01, 02, and 18, with cell viability of ≥ 53%, except for NLC + AMTAC 22, which showed values of less than 38%. When the US with standardized exposure was applied simultaneously with the high-concentration composition of NLC + AMTAC, the molecules’ cell viability decreased significantly compared to the DOX results (46.07%).

In an overview of the cell viability results and the SOSG assay, it can be predicted that AMTAC 02 may be the best choice of sonosensitizing compound since it shows significant toxicity to MCF-7 cells in the presence of US, as well as high SOSG fluorescence emission. In addition to showing promising cytotoxic activity against MCF-7 cells, the AMTAC 02 molecule was previously analyzed by Albino et al. for cytotoxicity in macrophages, and a CC_50_ of 569.50 μM was determined.^60^ Thus, AMTAC 02 was chosen to continue the *in vitro* release and *in vivo* antitumor activity tests (see details of the data obtained in Table S3).

Regarding the safety and selectivity of SDT and PDT, in addition to the selective activation of AS only with the action of US (or light, in the case of PDT), many proposals have been explored to understand the localization properties of sonicating agents and photosensitizers within tumors, in addition to the EPR effect. One of the most discussed mechanisms revolves around high cellular metabolism in the tumor region, which leads to a greater accumulation of these molecules in cancer cells than in healthy cells.^61,62^

High cellular metabolism requires a large amount of material to create membranes during cell division, for example. This role can be attributed to low-density lipoproteins (LDL), which provide the cholesterol necessary for this purpose, thus causing cells to absorb more LDL. As a result, some AS tend to interact with these proteins, which act as carriers to the tumor tissue.^61,62^

Additionally, the sonoluminescence mechanism proposed by SDT acts similarly to PDT, which leads to the belief that there is a possibility of disrupting the tumor’s vascular system, in addition to stimulating the immune system, resulting in occlusion of the tumor vessels and consequent accumulation of inflammatory cells at the affected site.^61,62^

### Release of AMTAC 02 from NLC Particles

The line equation calculated (Figure S2) to observe the linearity of the method and quantify the AMTAC 02 present in the NLC was equivalent to *y* = 50.383*x* + 52.944, determining a correlation coefficient value (*R* ^2^) of 0.9361. The limit of detection and quantification were 0.0618 and 0.1874 μM, respectively. The results showed that NLC + AMTAC 02 was linear in the concentration range of 2–8 μM, and the method was capable of detecting and quantifying low concentrations of the compound.

Figure 4 shows the *in vitro* release profile of AMTAC 02 (free) and NLC + AMTAC 02.

**Figure 4:**
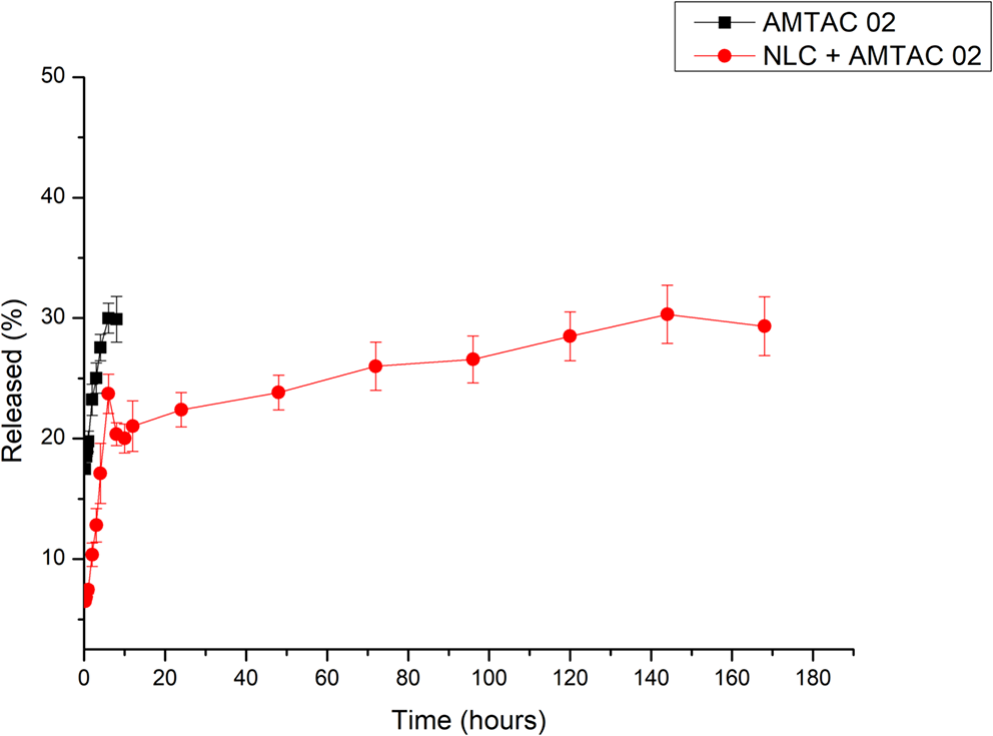
Release profile of pure free AMTAC 02 (black), and NLC + AMTAC 02 (red) incorporated into the release membrane. The results are expressed as the mean and standard deviation (SD) of *n* = 3.

In the first few minutes of release, the DMSO used to solubilize the AMTAC and present in the medium used was somewhat unstable, directly influencing the solutions. This may be due to the temperature of the location at which the samples were prepared (10 ± 2 °C). DMSO has a freezing temperature of around 18 °C and its defrosting can lead to an imbalance in its particles; therefore, it can take a few hours after defrosting for the particles to reorganize themselves properly. At high temperatures, DMSO is also thermally unstable, leading to its decomposition.^63,64^

Additionally, light intervention may have occurred at certain stages of the process. It is known that some spiroacridine derivatives can undergo photolysis, since the acridine nucleus is photoreactive, leading to the belief that AMTAC molecules may also exhibit phototoxicity when excited by light.^65,66^

Furthermore, considering this point and knowing that the mechanism of photodynamic therapy is based on the excitation of the photosensitizing agent from its singlet state to its triplet state, called intersystem crossing, some studies indicate that this transition causes structural changes in the molecule, altering reading parameters, such as a decrease in the reading signal, in addition to causing changes in the selected wavelength, thus hindering the detection of the molecule after the light activation mechanism. In cases like this, possible solutions can be addressed, such as the application of the HPLC reading technique, observing molecule degradation products, and changes in experimental conditions.^65,66^

Subsequently, for both systems tested, pure AMTAC 02 and NLC + AMTAC 02, AMTAC showed a certain instability when the 8-h test mark was reached, which can be seen in Figure 4, represented by a drop in the % release of the component. In the work carried out by Araujo, 2023, the results presented refer to N-acylhydrazone derivatives, which have similar behavior to AMTAC. In their work, the compound cited as JR-19 solubilized in DMSO was subjected to different temperatures and light cycles to observe its stability, yielding positive results in up to 8 h of testing. The paper then presents results relating to stability as a function of storage, providing information that at refrigerator temperature, the compound did not show stability, and once again, this instability may be related to the characteristics of DMSO.^63^

As for payload release percentage from the NLC AMTAC 02 particles within the dialysis membrane, the release profile of AMTAC 02 is modified, releasing around 30% of the payload within 144 h, the same amount that the free drug was able to release within 8 h. This characteristic is due to the behavior of nanostructured delivery systems. In this case, the phenomenon can be attributed to the shape of the composition of the imperfect NLC matrix, since, due to the disorganization of its crystals, there is a delay in the polymorphic transition, interfering with the release rate of the compound.^67,68^

Since the payload is very hydrophobic, the results suggest that it may exhibit a preference to remain in the NLC.^69,70^ This would ensure that the intact particle would be taken into the target cell, and this would ensure intracellular generation of ROS and a more effective therapeutic effect.^71,72^

### *In Vivo* Efficacy

To examine therapeutic efficacy *in vivo*, a human breast cancer tumor xenograft model (MDA-MB-468) in BALB/c mice was employed as described in the Methods section. The treatments were applied after the tumors reached an average volume of 65 mm^3^, and all the animals survived the procedure. Figure 5 shows the plot of tumor growth inhibition for the various groups.

**Figure 5:**
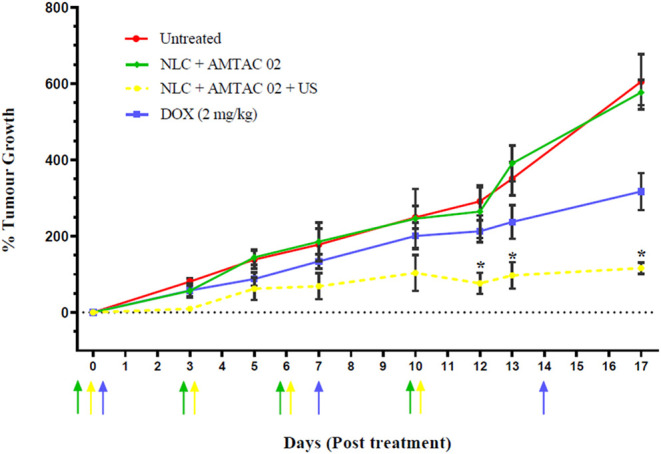
Profile of tumor growth in nude mice after treatment with the system containing NLC + AMTAC 02 (green), NLC + AMTAC 02 + US (yellow), negative control (red), and positive control (blue) according to time. Arrows represent the days of treatment. Results expressed as mean ± standard deviation and *n* = 5. *p < 0.05.

Following the same color patterns as the graph, the arrows represent the treatment days for each group: NLC + AMTAC 02 (green), NLC + AMTAC 02 + US (yellow), and DOX (blue). For control purposes, DOX was chosen as the reference treatment, since it is widely used in clinical treatment protocols for various types of cancer, including breast cancer, administered at a concentration of 2 mg/kg, which is slightly lower than what is normally administered to patients in these conditions.^7,73,74^

At day 7, treatment with DOX resulted in tumors growing to 133% of the original group starting volume, which compared favorably with the growth of the untreated control group, which had increased by 178% of the pretreatment volume. As for day 17, the group presented an increase of 317% from the initial volume, while the untreated group had a rise of 673%. As for the NLC + AMTAC 02 group, the formulation followed the tumor growth pattern of the untreated group, corroborating the results cited above, stating that US is a fundamental basis for the treatment to be applied.

After the last treatment with the NLC + AMTAC 02 + US formulation on day 10, significantly greater patterns of tumor growth inhibition were observed compared to the untreated group. Even with the highest reading percentages, on day 17, the formulation brought results of 116% tumor growth inhibition, a significantly lower average when compared to the other groups, with approximately 6 times more growth inhibition compared to the untreated group (*p < 0.05, ANOVA and Tukey Test).

Additionally, it is important to note that the cells implanted in the animals belonged to a triple-negative BC cell line, which has a poor prognosis when compared to other breast cancers, it is also one of the most aggressive types of cancer and is associated with a high recurrence rate, highlighting the pharmacological activity of the formulation.^75,76^ Furthermore, there was no significant variation in the weight of the animals, which showed a weight variation of ± 9%, showing a well-tolerated therapy, with possible reductions in adverse effects, leading us to believe the application of the formulation with concomitant use of US presents itself as a possible alternative for the treatment of BC.^75,76^

In this context, considering that the application of the system composed of NLC + AMTAC 02 + US was able to cause significant cell death in MCF-7 cells and significant tumor inhibition in MDA-MB-468 cells, these behaviors suggest that the fundamental mechanism of action of SDT can be applied and perform its role independently of the cell line analyzed, when correctly activated. This mechanism is based on the exacerbated formation of ROS through inertial cavitation activated by the interaction of a sonosensitizing agent, ultrasound, and molecular oxygen. Thus, its action is based on biophysical processes and not on genetic markers that are specific to cancer cells. However, the level of molecular oxygen present in the tumor, as well as the capacity for cell repair and the levels of antioxidant agents, may interfere with the response to SDT.^13^

This fact is important, since multidrug resistance (MDR) is one of the greatest challenges in the treatment of cancer, including BC, especially in advanced stages of the disease, or in recurrent cases and triple-negative breast cancer, as they can present, in some cases, overexpress efflux transporters, which actively expel the administered chemotherapy drugs; present changes in cellular metabolism and be capable of neutralizing some chemotherapy agents; in some cases, mutations or changes in the expression of target proteins may occur; they may even be capable of increasing the expression of antiapoptotic proteins.^77^ Thus, the application of SDT may be promising in these cases.

Further studies involving the mechanism of action proposed by SDT in this case, such as the application of techniques involving flow cytometry, basal cellular cytotoxicity, biological interaction studies, and *in vivo* acute toxicity studies, should be conducted to ensure greater safety of the compound.

## Conclusion

The system developed from the incorporation of AMTAC into NLC showed values of average diameter and PDI, as well as ZP characteristics of a homogeneously dispersed and physicochemically stable nanosystem, providing the system with the possibility of participating in the EPR effect, in addition to the other advantages presented. Based on the SOSG fluorescence emission tests, in which AMTAC 02 showed a higher rate of ROS generation when activated by US, and the MTT results, which resulted in low cell viability for MCF-7 BC cells, it was possible to select a specific AMTAC molecule to be applied to the system and thus continue the experiments.

A modified release profile was demonstrated by AMTAC 02 from the NLC, an expected characteristic due to the disorganized profile of the matrix in the nanosystem. The *in vivo* test carried out on animals with triple-negative BC MDA-MB-468 cells implanted showed a 6-fold higher rate of tumor growth inhibition when compared to the untreated group, as well as no significant variation in the animals’ body weight. These results describe a formulation that presents an attractive formulation as a delivery vehicle for AMTAC 02 for the application of SDT, presenting itself as a possible alternative treatment for BC.

## Supplementary Material



## Notes

Table S1: Description of the different forms of AMTAC, Figure S1: Visual appearance of NLC+AMTAC, Figure S2: Calibration curve, Table S2: Detailed results regarding cell viability (PDF)
