# Supplementary material for: Nanostructured Lipid Carriers Containing Acridine Derivatives in the Application of Sonodynamic Therapy for the Treatment of Breast Cancer
Source: ACS Omega. 2026 Feb 3;11(6):10231–41. doi: 10.1021/acsomega.5c11337 (PMC12917635; doi:10.1021/acsomega.5c11337)
Supplement: Supplementary file 1 [file ao5c11337_si_001.pdf]

NANOSTRUCTURED LIPID CARRIERS CONTAINING ACRIDINE DEVIRATES FOR THE APPLICATION OF SONODYNAMIC THERAPY IN THE TREATMENT OF BREAST CANCER

Kammila M. N. Costa[1], Mariana R. Sato[1], Ricardo O. de Moura[1,2], Anthony P. McHale[3], John F. Callan[3], João A. Oshiro-Junior[1,2]*

[1]Postgraduate Program in Development and Technological Innovation in Medicines, Federal University of Paraíba, João Pessoa, Paraíba, Brazil.

[2]Pharmaceutical Sciences Postgraduate Program, Center for Biological and Health Sciences, State University of Paraíba, Av. Juvêncio Arruda, S/N, Campina Grande 58429-500, PB, Brazil

[3]Ulster University – Coleraine, Northern Ireland, UK.

[*]Corresponding author: joaooshiro@yahoo.com.br

Supplementary information

Table S1. Descriptive table covering the different forms of AMTAC.

Compound	Molecular formula	Structural formula	Nomenclature	IC50 Value (µM)
AMTAC 01	$C_{24}H_{17}N_4O$	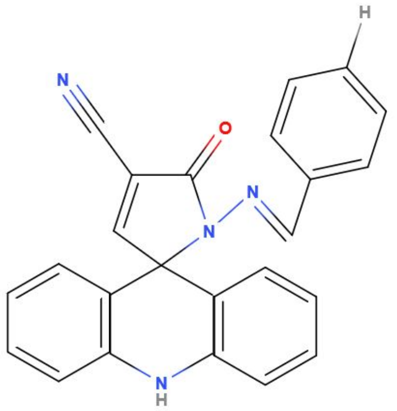	(E)-1´-(benzylideneamino)-5´-oxo-1´,5´-dihydro-10H-spiro[acridine-9,2´-pyrrole]-4´-carbonitrile	70.54
AMTAC 02	$C_{25}H_{20}N_4O_2$	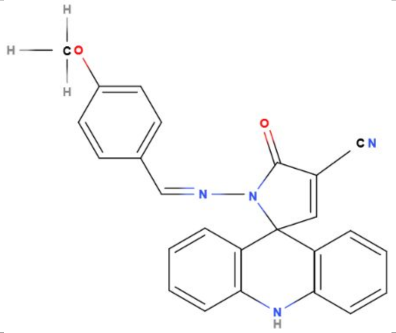	(E)-1´-((4-methoxybenzylidene)amino)-5´-oxo-1´,5´-dihydro-10H-spiro[acridine-9,2´-pyrrole]-4´-carbonitrile	75.43

AMTAC 18	$C_{26}H_{22}N_4O_3$	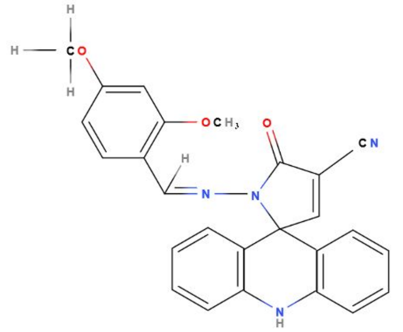	(E)-1'-((2,4-dimethoxybenzylidene)amino)-5'-oxo-1',5'-dihydro-10H-spiro[acridine-9,2'-pyrrole]-4'-carbonitrile	72.87
AMTAC 22	$C_{26}H_{19}N_5O$	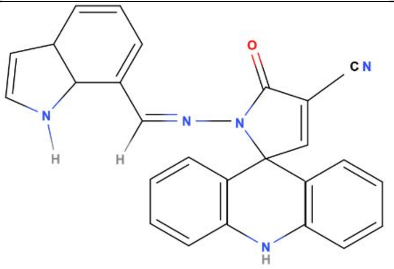	1'-(((1H-indol-7-il)methylene)amino)-5'-oxo-1',5'-dihydro-10H-spiro[acridine-9,2'-pyrrole]-4'-carbonitrile	40.82

Figure S1. Visual aspect of nanostructured lipid carriers containing AMTAC.

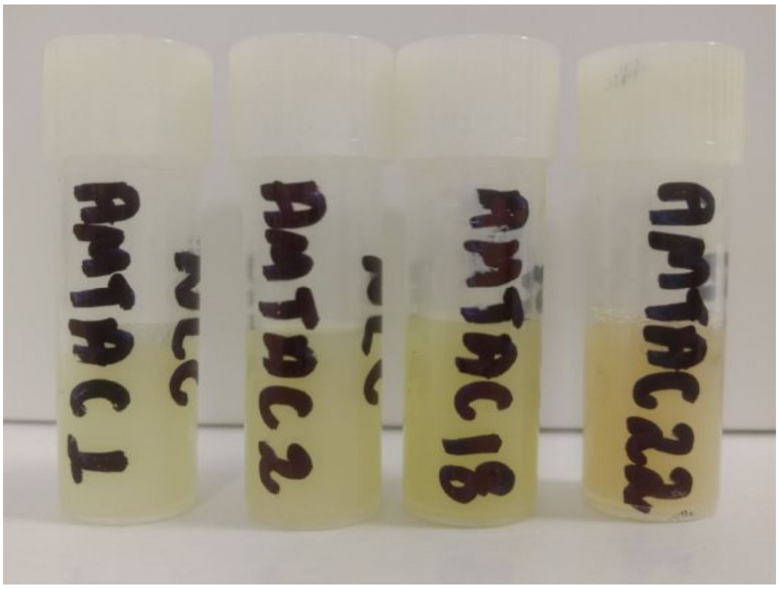

Figure S2. Calibration curve of AMTAC 02.

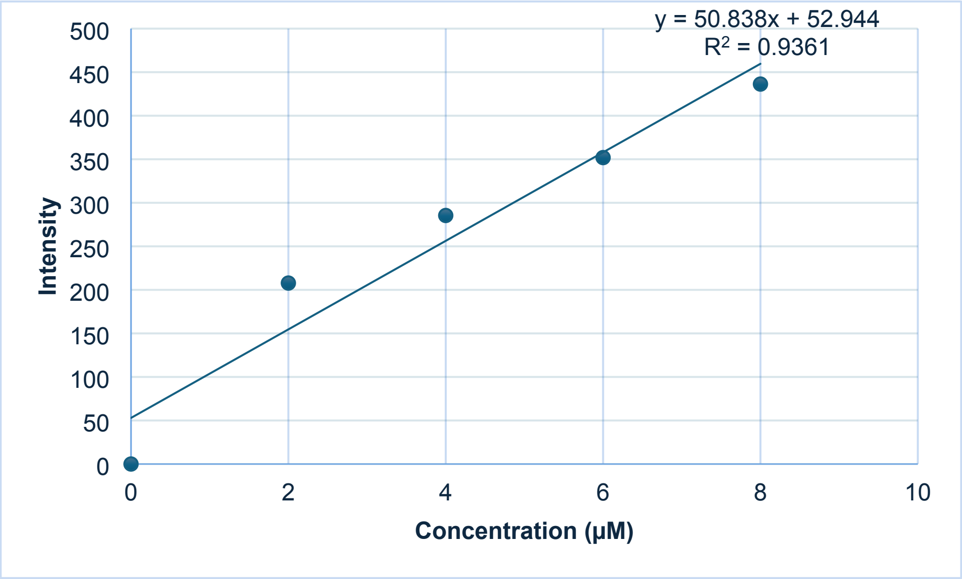

Note: Dimethylsulfoxide (DMSO); Phosphate buffer (PBS).

Table S2. Cell viability data of MCF-7 breast cancer cells after treatment with the system containing NLC and different forms of AMTAC compared to the control. Results are expressed as mean ± SD for n = 4.

Compound (AMTAC µM)	US	Viability cell N1 (%)	Viability cell N2 (%)	Viability cell N3 (%)	Viability cell AVERAGE (%)
NLC blank	-	80.70 ± 8.06	68.56 ± 3.05	83.10 ± 7.10	79.64 ± 8.64
NLC blank	30s	74.01 ± 3.58	77.46 ± 8.14	67.56 ± 3.09	73.01 ± 4.01
DOX 46 Control	-	44.67 ± 3.70	46.57 ± 5.71	47.00 ± 5.16	46.07 ± 1.01
DMEM Control	-	107 ± 9.60	90.66 ± 7.41	100.66 ± 6.77	96.36 ± 4.23
NLC + AMTAC 01 – 6.6	-	103.84 ± 13.73	97.67 ± 3.29	87.18 ± 5.90	96.23 ± 6.87
NLC + AMTAC 01 – 13.2	-	98.18 ± 6.24	97.60 ± 6.59	77.35 ± 7.01	91.04 ± 9.68
NLC + AMTAC 01 – 66	-	47.73 ± 10.25	55.29 ± 4.73	56.88 ± 6.91	53.30 ± 3.99
NLC + AMTAC 01 – 66	30s	36.63 ± 3.63	38.35 ± 3.89	25.76 ± 4.90	28.57 ± 7.15

NLC + AMTAC 02 – 6.6	-	90.66 ± 6.08	106.04 ± 2.52	98.00 ± 5.09	98.23 ± 6.28
NLC + AMTAC 02 – 13.2	-	82.46 ± 1.31	92.94 ± 2.25	86.06 ± 4.05	87.15 ± 4.34
NLC + AMTAC 02 – 66	-	44.81 ± 7.59	64.83 ± 8.39	60.09 ± 5.58	56.57 ± 8.54
NLC + AMTAC 02 – 66 + US 30s	30s	36.63 ± 3.63	38.35 ± 3.89	25.76 ± 4.90	33.58 ± 5.57
NLC + AMTAC 18 – 6.6	-	84.23 ± 7.22	94.98 ± 4.62	79.21 ± 9.09	86.14 ± 6.57
NLC + AMTAC 18 – 13.2	-	79.39 ± 4.04	92.69 ± 9.43	71.97 ± 1.02	81.34 ± 8.57
NLC + AMTAC 18 – 66	-	59.05 ± 5.75	56.15 ± 6.27	46.08 ± 8.22	53.76 ± 5.55
NLC + AMTAC 18 – 66	30s	32.98 ± 3.95	33.10 ± 2.78	28.81 ± 5.63	31.63 ± 1.99
NLC + AMTAC 22 – 6.6	-	76.01 ± 9.70	77.46 ± 4.56	75.83 ± 3.84	76.64 ± 0.81
NLC + AMTAC 22 – 13.2	-	62.29 ± 5.53	50.19 ± 5.37	50.77 ± 3.67	54.41 ± 5.57
NLC + AMTAC 22 – 66	-	31.93 ± 3.49	38.56 ± 3.58	43.57 ± 3.08	38.02 ± 4.76
NLC + AMTAC 22 – 66	30s	34.71 ± 4.79	39.04 ± 0.86	34.88 ± 7.73	36.21 ± 2.00

Note: Nanostructured lipid carrier (NLC); Ultrasound (US); N1, N2, N3 (triplicates); Thirty seconds (30s).
